# Behavioural symptoms in patients with Alzheimer's disease and their association with cognitive impairment

**DOI:** 10.1186/1471-2377-10-87

**Published:** 2010-09-28

**Authors:** Manuel Fernández, Ana L Gobartt, Montse Balañá

**Affiliations:** 1 Servicio de Neurología. Hospital de Cruces, Plaza de Cruces s/n 48903 Barakaldo, Spain; 2 Departamento Médico, Novartis Farmacéutica S.A., Gran Via de les Corts Catalanes 764, Barcelona, Spain

## Abstract

**Background:**

Behavioural and psychological symptoms of dementia (BPSD) are non-cognitive symptoms commonly associated to Alzheimer's disease (AD). The characterization of the clinical profile of AD patients might help to better understand disease evolution and to improve diagnosis and treatment. Thus, the aim of the present study is to describe the clinical profile of AD patients, and to correlate the presence of BPSD with the severity of the disease.

**Methods:**

A cross-sectional, observational and multicenter study was conducted at 115 centres in Spain. Patients suffering from AD with higher and lower BPSD scores (ADAS-Noncog score 26-50 and ≤25, respectively) were included. Demographic and clinical data were collected, and dementia severity was assessed by the Mini Mental State Examination (MMSE) [mild 27-21, moderate 20-11, severe ≤10]. The use of ADAS-Noncog in clinical practice was also explored.

**Results:**

A total of 1014 patients (463 with higher and 551 with lower BPSD scores) were included (mean age 77 ± 7 years, 65% women). Almost all patients (90%) had BPSD at inclusion, 17% of which reported psychotic outbreaks. The most prevalent symptoms were lack of concentration (56%), tremors (56%), depression (44%), lack of cooperation (36%), and delusions (32%). Patients with higher BPSD scores showed a significantly higher prevalence of psychotic symptoms (delusions, hallucinations, and delirium) and tremors, while emotional symptoms (tearfulness and apathy) predominated in patients with lower BPSD scores. MMSE and ADAS-Noncog scores were negatively associated (p = 0.0284), suggesting a correlation between cognitive impairment and BPSD. Lack of concentration and appetite change significantly correlated with MMSE (p = 0.0472 and p = 0.0346, respectively). Rivastigmine and donepezil were the first choice therapies in mild to moderate dementia. ADAS-Noncog was generally considered better or similar to other scales (82%), and 68% of the investigators were willing to use it in the future.

**Conclusions:**

Our study shows that patients with AD have a high prevalence of noncognitive symptoms, and that cognitive impairment and BPSD are correlated. Therefore, ADAS-Noncog is a useful evaluation tool.

## Background

Dementias, and particularly Alzheimer's disease (AD), are a growing public health problem that results from population ageing [1]. The hallmarks of dementia are functional, cognitive, and behavioural manifestations.

Among the first manifestations of dementing disorders are behavioural symptoms which might appear before cognitive alterations [2], at some time during the course of the illness [2,3] and vary according to dementia severity [4].

Behavioural and psychological symptoms of dementia (BPSD) are non-cognitive symptoms commonly associated to AD. Early detection of BPSD is extremely important, because these symptoms not only induce noticeable disability in demented patients, but also increase the caregiver stress [5]. In fact, BPSD increase impairment in daily living activities [6,7], accelerate cognitive decline [8] and worsen patient's quality of life [9]. Besides, behavioural disturbances represent the main reason for and lead to early patient institutionalization [10,11], increasing the overall financial cost [12]. However, when correctly diagnosed, these disorders can be efficiently treated with drugs [13,14] and, therefore, delay nursing home placement and improve patients' and caregivers' quality of life.

Several studies have been conducted to assess the prevalence of BPSD symptoms, so depending on the type of population studied and on the methods used to assessed it, the prevalence ranges from 61% to 92% [15-18]. Since the frequency of BPSD is highly variable, the correct assessment of these symptoms may contribute to a better diagnosis and treatment, and could be a powerful tool to evaluate the efficacy of any therapy directed towards the improvement of behavioural disturbances.

Currently, BPSD can be assessed by standardized instruments such as the Neuropsychiatric Inventory (NPI) [15,18,19], the Behaviour Pathology in Alzheimer's Disease (BEHAVE-AD) scale [20], and the Alzheimer's Disease Assessment Scale (ADAS) [21,22]. The ADAS, and especially its non-cognitive subscale (ADAS-Noncog), is a specific diagnostic tool designed to detect BPSD in patients with AD. An adapted and validated Spanish version of this rating scale is also available [23,24].

A cross-sectional and multicenter study has been conducted with the aim of describing the BPSD and the clinical profile (age, gender, severity of dementia) of AD patients showing lower and higher behavioural symptoms, according to the ADAS-Noncog scale. In addition, the relationship between BPSD and cognitive impairment severity of was assessed.

Secondary endpoints were to describe extrapiramydal symptoms in AD patients, to compare Mini Mental State Examination (MMSE) scores in patients with higher and lower BPSD, to analyse the relationship between MMSE scores and BPSD, and to describe the different behavioural syndromes observed in different subsets of patients.

## Methods

### Study design and conduct

A cross-sectional, observational, and multicenter study was conducted at 115 neurology centres in Spain. The study was approved by the Clinical Ethics Committee of the Hospital de Cruces (Bilbao), and all patients or their legal representatives provided written informed consent. The study was in accordance with the Helsinki Declaration principles for medical research involving human subjects.

### Patient population

Patients with diagnosis of AD according to DSM-IV criteria and with available clinical data from the previous year were eligible for the study. During five months, each physician recruited five to six patients with higher BPSD scores and five to six patients with lower BPSD scores. Patients in the first group had ADAS-Noncog total scores ranging from 26-50 and patients in the lower group had scores ≤25. The ADAS-Noncog is a validated 10-item tool that examines aspects of mood (tearfulness and depression) and behaviour (lack of concentration, lack of cooperation, delusions, hallucinations, pacing, motor behaviour, tremors, and appetite change). Each item is rated on a 5-point scale; thus, total scores range from 0 to 50. Higher scores represent greater mood or behavioural disturbances. Ratings were made by the examiner based on the information obtained from patients, family members or caregivers, and professional observations.

At the selection visit, in addition to ADAS-Noncog test, MMSE test was performed and the presence or absence of BPSD during the last year was reported.

### Variables

The main outcome of the study was the ADAS-Noncog score obtained at baseline. The main variable of the study was dementia severity which was categorized into three categories: mild (27-21), moderate (20-11) and severe (≤10) according to MMSE scores.

Secondary variables included demographic data, years since AD diagnosis, pharmacological treatments for AD/BPSD in the last year, concomitant diseases and treatments, presence of psychotic breaks and extrapyramidal symptoms (tremors, rigidity, hypokinesia, postural instability, and fluctuating cognition) during the last year, and the description of the most frequent BPSD at inclusion and during the previous year (possible symptoms included ADAS-Noncog items and some BPSD of the International Psychogeriatric Association [25], such as apathy, anxiety, agitation, and delirium). Finally, physicians were asked about their use of the ADAS-Noncog scale in clinical practice.

### Statistics

Sample size calculation was based on the description of dementia severity and was calculated using NCSS 2004 and PASS 2002 softwares. A minimum of 507 patients per BPSD group were required to obtain a precision of 4.5% for each dementia degree with a 95% confidence interval (95%CI), assuming a 10% of patient losses.

All statistical analyses were made with the SAS 8.2 software using a two-tailed alpha level of 0.05. Data are presented as percentages for discrete variables, and as mean ± SD for continuous variables. Differences between groups were assessed using the Chi-square test for categorical data and using one-way ANOVA (or Mann-Whitney test when data was not normally distributed) for continuous variables. Finally, the association between continuous variables was tested by using the Spearman correlation coeficient. The strength of the association was regarded as very weak for Spearman coefficient values of 0-0.19, as weak for 0.2-0.39 values, as moderate for 0.40-0.59 values, as strong for 0.6-0.79 values, and as very strong for 0.8-1 values. Principal component analysis was used for factor analysis of the ADAS-Noncog. Factors were selected on the basis of eigenvalue-one criterion and varimax (orthogonal) rotation was applied.

## Results

### Population description

A total of 1014 patients were included in the study, 463 of them were in the higher BPSD group and the remaining were in with the lower BPSD group. One hundred and sixty-one additional subjects were excluded from the analysis due to absence of ADAS-Noncog score and/or not meet inclusion criteria. The mean ADAS-Noncog score in the in the higher BPSD group was 29.8 ± 3.4, indicating a moderate degree of behavioural symptoms. Patients in the lower BPSD group showed an average score of 10.4 ± 6.4. A summary of the most relevant characteristics of the studied population are presented in Table 1.

**Table 1:** Demographic and clinical history data of patients with AD with higher (N = 463) and lower BPSD scores (N = 551).

	AD + BPSD	AD - BPSD
Age (mean ± SD)	77.3 ± 6.7	77.1 ± 6.9

Gender (% men)	35.4%	34.8%

Years since AD symptoms (mean ± SD)	3.87 ± 2.32	3.89 ± 2.50

Years since AD diagnosis (mean ± SD)	1.32 ± 1.33	1.26 ± 1.19

Prior diagnosis of BPSD (before1 year)	42.7%	39.1%

Current treatment for AD	94.8%	95.6%

Behavioural symptoms (last year)	89.8%	89.7%

Tearfulness	36.1%	40.8%

Depression	49.9%	51.9%

Concentration	59.0%	59.7%

Cooperation	40.0%	42.5%

Delusions	42.1%	37.0%

Hallucinations	32.8%	28.5%

Pacing	31.5%	26.5%

Motor activity	35.2%	33.8%

Appetite change	29.8%	29.6%

Apathy	23.8%	24.0%

Anxiety	27.6%	24.5%

Agitation	25.5%	24.1%

Delirium	8.9%	6.9%

Extrapyramidal symptoms (last year)	53.8%	54.6%

Tremors	37.6%	38.3%

Rigidity	14.0%	14.3%

Hypokinesia	11.2%	9.8%

Postural instability	9.9%	8.3%

Fluctuating cognition	8.6%	7.6%

Psychotic breaks (last year)	18.4%	16.0%

N° breaks (mean ± SD)	2.43 ± 3.58	1.74 ± 1.68

Dementia severity: MMSE Severe	12.8%	15.1%

Moderate	46.5%	46.6%

Mild	40.7%	38.3%

AD + BPSD: patients with higher ADAS-Noncog scores. AD - BPSD: patients with lower ADAS-Noncog scores.

The mean age of the overall sample was 77.2 ± 6.8 years old, and 65% were women (Table 1). Half of the patients (51%) had no primary education, whereas 37% of them completed primary school, 9% secondary education, and 3% had a university degree. Since most patients (85%) were living at their home, the caregiver was usually a family member, especially a son or daughter (54%) or the spouse (39%). Only the 3% of caregivers were paid medical professionals.

As described in Table 1, 90% of patients had shown behavioural symptoms during the previous year. The most common were lack of cooperation and depression, with prevalence above 50%. It is noteworthy that these symptoms had been underdiagnosed, since only 41% of the studied population had prior diagnosis of BPSD. Likewise, more than half of the patients (54%) had experienced extrapyramidal symptoms, predominantly tremors (38%) and rigidity (14%). Psychotic breaks occurred to 17% of patients, with an average of 2.1 ± 2.7 breaks.

On the basis of the MMSE score, dementia severity was usually mild (39%) to moderate (47%). Only the 14% of the patients had severe dementia (Table 1).

### Clinical profile according to the scores BPSD

According to the results described in Table 1, no significant differences were observed between both groups of patients regarding age, gender, duration of AD, and treatment. The occurrence of BPSD, extrapyramidal symptoms, and psychotic breaks, during the previous year was also comparable between groups.

At inclusion time, almost all patients showed some behavioural symptoms (92%). The most prevalent were: lack of concentration (56%), tremors (56%), depression (44%), lack of cooperation (36%), delusions (32%), motor behaviour (29%), and tearfulness (28%) (Table 2).

**Table 2:** Description of current behavioural symptoms in patients with AD with higher (N = 463) and lower BPSD scores (N = 551).

	AD + BPSD	AD - BPSD
Behavioural symptoms (at inclusion)	92.2%	91.7%

Tearfulness	24.6%	31.4%*

Depression	44.5%	43.6%

Concentration	56.4%	55.4%

Cooperation	34.6%	37.0%

Delusions	35.2%	29.2%*

Hallucinations	27.0%	21.6%*

Pacing	26.6%	21.4%

Motor activity	29.4%	27.9%

Tremors	80.6%	34.2%***

Appetite change	28.5%	24.3%

Apathy	17.1%	22.5%*

Anxiety	20.3%	17.4%

Agitation	15.3%	18.3%

Delirium	4.8%	2.2%*

AD: Alzheimer's disease; BPSD: Behavioural and psychological symptoms of disease; AD + BPSD: patients with higher ADAS-Noncog scores. AD - BPSD: patients with lower ADAS-Noncog scores; Chi-square test: *p < 0.05; ***p < 0.001.

Symptoms which significantly contributed to characterize the patients in the higher BPSD group were psychotic, such as delusions (p = 0.0417), hallucinations (p = 0.0451), delirium (p = 0.0233), and tremors (p < 0.001). Among patients in the lower BPSD group, the most prevalent were emotional symptoms such as tearful mood (p = 0.0170) and apathy (p = 0.0310). The frequencies of all BPSD between groups at inclusion are compared in Table 2.

Regarding dementia severity, there were no significant differences in MMSE score between both groups (p > 0.05).

### Correlation between the presence of BPSD and dementia severity

A significant negative association was observed between dementia severity (MMSE score) and ADAS-Noncog score (p = 0.0284) in patients in the higher BPSD group, indicating that BPSD symptoms got worse when cognition decreased. Specifically, the behavioural symptoms which showed significant associations with MMSE were lack of cooperation (p = 0.0472) and appetite change (p = 0.0346). In regard to, lack of concentration only a tendency was observed (p = 0.0580). Detailed results of correlation analyses are provided in Table 3.

**Table 3:** Description of current behavioural symptoms in patients with AD with higher (N = 463) and lower BPSD scores (N = 551).

Correlation between dementia severity (MMSE) and ADAS-Noncog score		
	Present MMSE score	

	Spearman correlation coefficient	P-value

Total ADAS-Noncog score	-0.10168	0.0284

Correlation between ADAS-Noncog items and dementia severity (MMSE)		

	Present MMSE score	

	Spearman correlation coefficient	P-value

Tearfulness	0.04663	0.3167

Depression	0.01318	0.7775

Concentration	-0.08797	0.0580

Cooperation	-0.09210	0.0472

Delusions	-0.01221	0.7928

Hallucinations	-0.04601	0.3237

Pacing	-0.05000	0.2841

Motor activity	-0.04709	0.3115

Tremors	-0.02424	0.6037

Appetite change	-0.09822	0.0346

### Alzheimer's disease treatment

Almost all the evaluated patients (94%) were treated with antidementia drugs: rivastigmine (47%), donepezil (24%), or memantine (23%) as mono or combined therapy for the previous year. Besides, 37% of patients took antipsychotic drugs, mostly risperidone (21%). Patients with extrapyramidal symptoms received significantly more risperidone (15%) than patients without extrapyramidal symptoms (6%) [p < 0.001].

When treatments were stratified by disease severity, rivastigmine and donepezil were the first choice therapies among patients showing either mild or moderate dementia, while memantine was the most prescribed drug in patients with severe AD. In contrast, no significant differences in the use of cholinesterase inhibitors and memantine were found in regard to the presence of BPSD or extrapyramidal symptoms.

### Use of ADAS-Noncog in clinical practice

In physician's opinion, ADAS-Noncog was generally considered better than (47%) or similar to (35%) other scales. An important percentage of the investigators regularly used an instrument for assessing BPSD (65%). Among the tools used to assess BPSD stood out the NPI (50%) and the ADAS-Noncog scale (28%), whereas other scales, such as BEHAVE-AD, Caretaker Obstreperous-Behaviour Rating Assessment (COBRA), or Blessed Dementia Scale (BDS), were used by less than 2% of physicians. Finally, among the physicians who were willing to use the ADAS-Noncog in the future (68%), 97% were already using it (Spearman's correlation, p < 0.0001).

### Factor analysis

The results of the factor analysis in both, patients with the lower and the higher BPSD scores are shown in Tables 4 and 5. The ten BPSD symptoms were explained by three and four factors, for the lower and the higher BPSD groups, respectively.

**Table 4:** Factor analysis of the ADAS-Noncong in patients with AD with lower BPSD scores (N = 551)

Factor analysis in patients with lower BPSD			
	**Factor 1**	**Factor 2**	**Factor 3**

Tearfulness	0.02642	*0.83697*	0.10166

Depression	0.13897	*0.82174*	0.06316

Concentration	0.53240	0.33285	0.15738

Cooperation	0.59021	0.18935	0.17487

Delusions	0.15631	0.13834	*0.82285*

Hallucinations	0.09756	0.00094	*0.84430*

Pacing	*0.75853*	0.03146	0.23335

Motor activity	*0.79410*	0.00864	0.23449

Tremors	0.52143	0.04723	-0.14602

Appetite change	0.39204	0.39735	-0.07791

Italics indicate highest factor loadings.

**Table 5:** Factor analysis of the ADAS-Noncong in patients with AD with higher BPSD scores (N = 463)

Factor analysis in patients with higher BPSD				
	**Factor 1**	**Factor 2**	**Factor 3**	**Factor 4**

Tearfulness	-0.16880	-0.12826	*0.76502*	-0.05303

Depression	-0.23948	-0.11968	*0.80061*	-0.02236

Concentration	0.06765	-0.09714	0.01880	*0.85541*

Cooperation	-0.01898	-0.03624	-0.08582	*0.85437*

Delusions	-0.05105	*0.77250*	-0.16114	-0.04323

Hallucinations	-0.10889	*0.76703*	-0.22915	-0.08983

Pacing	*0.86097*	-0.02711	-0.17152	0.01532

Motor activity	*0.86506*	-0.02129	-0.17001	0.03530

Tremors	-0.32619	-0.34570	-0.34536	-0.30184

Appetite change	-0.20893	-0.53308	-0.36340	0.04134

Italics indicate highest factor loadings.

In patients with the lower BPSD scores, three factors explained 56% of the data total variance. The first factor, which explained 31% of total variance, denoted a dimension representing hyperactivity and had the highest loadings on pacing and motor activity. The second factor, which explained 14% of total variance, represented an affective dimension and had the highest amount on tearfulness and depression. The third factor, which explained 12% of the total variance, represented a psychosis dimension and had the highest degree on hallucinations and delusions.

In patients with the higher BPSD scores, a new dimension was added to the three mentioned above. The four factors explained 66% of the data total variance in the data. The first factor, which explained 22% of the total variance, represented a hyperactivity dimension with the highest loadings on pacing and motor activity. The second factor, which explained18% of the total variance, represented a psychosis dimension and had the highest amount on hallucinations and delusions. The third factor, which explained 14% of the total variance, represented an affective dimension and had the highest degree on tearfulness and depression. Finally, the fourth factor, which explained 12% of the total variance, denoted a dimension represented by lack of concentration and cooperation.

## Discussion

ADAS-Noncog is a diagnostic tool especially suitable in outpatient clinics because it does not need a special training and its use requires a short time. In fact, its simplicity could be the main reason for the high proportion (68%) of participating physicians who reported willingness to use it in the future.

An additional advantage of ADAS-Noncog Scale lies in the interview-based assessment of the behavioural symptoms. In contrast, other scales exclusively based on patient relatives or caregiver's reports may distort symptoms, which could be objectively analyzed by clinicians.

The results obtained in the present study are consistent with other published investigations which demonstrate that lack of concentration, tremors, depression, lack of cooperation, and psychotic symptoms, are frequent in AD patients [26-30].

In our patient series, 90% of the overall population showed at least one BPSD in the previous year, and 92% at the time of the initial evaluation. Similarly, another study based on ADAS-Noncog scale [28] reported that 97% of all patients had some behavioural symptoms at admittance.

Acording to our results, the most frequent BPSD was lack of concentration and tremors with prevalence around 56%. Similar results were obtain by Marin and colleagues when studying non-cognitive disturbances in AD [28]. Concentration alterations and lack of attention appear very early in AD patients [31], probably due to the bilateral tempo-parietal degeneration associated to this disease [32]. Aside from its high prevalence, concentration decrease tends to be positively correlated with cognitive impairment, suggesting that this symptom should be considered a central deficit in AD. Tremor, in its different manifestations, is common among elderly population aged over 65 [33-35]. In our work, tremor was one of the behavioural symptoms that best differentiated between patients with higher and lower BPSD scores. In that regard, patients in the higher BPSD group had higher prevalences of delusions, hallucinations and delirium, so they had higher use of neuroleptic drugs and these ones would in turn induce or worsen tremors.

Among BPSD symptoms depression is also common in AD patients, and strongly correlates with tearful mood. In fact, in our study, depression occupied the third position with a prevalence of 44%. These results are in accordance with those obtained in previous studies [28,36-38]. However, depression frequencies are variable [39,15]. Although the relationship between dementia and depression is controversial, the latter constitutes an independent risk factor for AD [40]. Depressive symptoms are frequent among patients with AD in absence of major depression [41]. Similarly to ours, other studies did not found correlation between cognitive impairment and depression [42], indicating that the onset of depression might occur at any stage of the disease.

Lack of cooperation, which is generally related to an increased motor behaviour, may be a consequence of the normal course of disease. As for our results, the lack of cooperation is low correlated with the degree of cognitive impairment (MMSE score). However, its prevalence in our sample (36%) points out its relevance among noncognitive symptoms.

The proportion of patients showing increased motor activity or pacing (29% and 24%, respectively) is comparable to the one obtained in other studies [28,43]. Pacing is especially annoying to caregivers [44] and, depending on the population series, its occurrence range from 10% to 60% in AD patients [29,43,45,46]. Aberrant motor behaviour, which can lead to psychomotor agitation, is frequent in patients with AD [29], and represents the 38 out of 100 patients [19]. In our study, agitation reached a prevalence of 17%. Agitation may indicate a frontal affectation and has a deep impact in caregivers [47].

Psychotic symptoms of AD, including delusions and hallucinations, significantly contribute to characterize those patients in the higher BPSD group, probably because the increased severity of disease. Although previous studies demonstrate the relationship between delusions and hallucinations and the degree of cognitive impairment [48], no correlation was found in the present study. Both symptoms are predictors of an increased functional and cognitive decline. Moreover, hallucinations have been associated with a higher percentage of institutionalization and mortality [49]. In a cross-sectional study which included patients with AD, vascular dementia, and dementia with Lewy bodies (DLB), the presence of delusions and hallucinations were related to a higher agitation and aggressiveness [50]. Conversely, other studies failed to find any significant correlation [19].

Among behavioural symptoms, appetite change exhibited mild, although significant, negative correlation with dementia severity. Anorexia, hyperphagia, and dietary changes are some of the of the appetite disorders occurring in AD [5,44,46]. However, hypophagia is the most frequent alimentary disturbance in these patients. A previous study [51] demonstrated higher frequency and severity of BSPD in patients with AD who lost weight. Several hypotheses, such as medial temporal cortex atrophy, have been postulated to explain the hypophagia and weight loss observed in these patients [52].

The prevalence of apathy (20%) is similar to that reported in the literature [15]. Apathy is common among patients with AD, and has an early onset in the course of the disease [3,29,53]. A comparative study including patients with AD, patients with depression, and healthy subjects, showed that apathy may be found isolated or coexist with depression, but it does not increase depression scores [26].

Anxiety, involves the 19% of the studied patients. It predominates during the initial phases of the disease, when patients become aware of their deficit [54]. According to various studies, the frequency of anxiety in patients with AD ranges from 40% to 60%[19,55]. However, only 5% to 6% of the affected patients fulfil DSM-IV criteria for generalized anxiety disorder diagnosis [56].

In our patient population, a low significant association between dementia severity and total ADAS-Noncog score was found. Thus, according to our results, noncognitive symptoms might occur at any stage of AD and do not necessarily increase with dementia severity. Among behavioural symptoms, appetite disturbances and lack of cooperation showed the highest correlations, whereas a tendency was observed for concentration alterations. The rest of behavioural symptoms alone do not show significant correlations, so they have similar prevalence at any stage of disease. The relationship between cognitive impairment degree and BPSD is controversial, so that several studies supported it [43,57], while others do not [58]. Discordances in BPSD prevalence reported by different studies are probably due to the different scales used, as well as to cultural differences [59].

Regarding therapy, first choice treatments among patients with mild to moderate stages of the disease were administered rivastigmine and donepezil, whereas memantine was predominant in patients with severe dementia. The most used neuroleptic agent was risperidone. These data are similar to those provided by a recent study [60] which analyzed the use of psychoanaleptic drugs in our environment.

According to the data obtained in principal component analisys using the criterion of eigenvalues >1, it is possible to identify three different subsyndromes (hyperactivity, psychosis and affective dimension) in both, the higher and the lower, BPSD populations. Besides, patients with higher BPSD scores show a new subsyndrome, represented by the lack of concentration or cooperation. To the best of our knowledge, this new subsyndrome have been not previously described, and could be identified as an "inattentional syndrome" characterized by the loss of patience, the frustration, and the lack of cooperation induced by deficient attention. The present study also provides an additional evidence for the presence of neuropsychiatric subsyndromes in dementia, which were previously reported by studies using the NPI [61].

The present study shows some limitations that should be addressed. First of all, the patients sampled may suffer from more behavioural disturbances than the average population because the study was conducted in outpatient clinics. Second, the previous use of antidementia and psychotropic agents in our sample could affect the frequency, severity, and total scores of BPSD. Finally, the lack of correction for multiple testing is also a limitation of our study.

The frequency of hallucinations in our study is around 30%,, a percentage somewhat higher than that reported with the NPI in both epidemiological [15,36] and clinical populations [18,19]. Although this figure only indicates the presence of hallucinations in the last year, it does raise the possibility that there may have been a number of patients with DLB in our study population, particularly since many DLB patients could meet DSM-IV criteria for AD. Then, the exclusive use of DSM-IV criteria should also be adressed as a limitation.

From a qualitative perspective, the present results should be similar to those obtained in other studies in which different evaluation tools are used. In fact, although other tools [19,55] probably assess noncognitive symptoms in greater detail, ADAS-Noncog scale include the main behavioural symptoms for assess dementia. Nonetheless, ADAS-Noncog scale has the limitation of being scored by the examiner based upon patient and caregiver interview, and examiner impression. Consequently, the examiner might be overly influenced by his/her snapshot impression of the patient at the time of the visit, which may not be representative of patient's behaviours during typical daily routines.

In conclusion, the present work has several implications for the treatment of AD. The high prevalence of BPSD in patients suffering from AD implies that the assessment of behavioural symptoms is of great importance in clinical practice. In this regard, the use of scales such as ADAS-Noncog, which provide information from the patient and/or the caregiver in a short period of time, is recommendable in outpatient clinics.

BPSD are often a manifestation of cognitive decline and constitute one of the most common causes of family burden and patient institutionalization. The proper management of these symptoms will therefore increase their wellbeing and quality of life [62].

## Conclusions

The present study shows a high prevalence of BPSD in AD, since almost all patients (92%) showed some mood or behavioural symptom at the time of exploration. The most important frequent symptoms were lack of concentration (56%), tremors (56%), depression (44%), lack of cooperation (36%), delusions (32%), tearfulness (28%), and increased motor activity (29%). An "inattentional syndrome" was also identified in patients with the higher BPSD scores. Besides, a low correlation between the severity of cognitive impairment and the behavioural disturbances has been found. A total of 68% of physicians were willing to use the ADAS-Noncog scale in the future.

## Abbreviations

AD: Alzheimer's disease; ADAS-Noncog: Alzheimer's disease assessment scale (noncognitive subscale); BPSD: Behavioural and psychological symptoms of dementia; MMSE: Mini Mental State Examination.

## Competing interests

This study was funded by Novartis Farmacéutica S.A.

M. Fernández has no conflicts of interest to disclose. Ana L Gobartt and Montse Balañá are employees of Novartis Farmacéutica S.A. The funding source contributed to the study conception and design, and to the acquisition, analysis and interpretation of data.

## Authors' contributions

MF: main investigator, conceived of the study, and participated in its design and coordination.

ALG: co-investigator; participated in its design and coordination.

MB: co-investigator; participated in its design and coordination.

The article is not under consideration elsewhere and it has been read and approved by all listed authors.

## Pre-publication history

The pre-publication history for this paper can be accessed here:

http://www.biomedcentral.com/1471-2377/10/87/prepub
